# Is it safe? Health promotion videos on YouTube and the safety of viewers – Views from Ghana

**DOI:** 10.1016/j.pmedr.2024.102836

**Published:** 2024-07-22

**Authors:** Martin Gameli Akakpo, Evelyn Owusu Roberts

**Affiliations:** Department of Human Development and Psychology, Faculty of Arts and Sciences, Regent University College of Science and Technology, P.O. Box DS 1636, Accra, Ghana

**Keywords:** YouTube, Health promotion, Patient-caregiver communication, Patients, Caregivers, Health literacy

## Abstract

**Objectives:**

Health promotion videos are trending and abundant. Information provided in these videos is not verified by any designated experts but is popular. In this paper we discuss the prevalence of such videos and guide patients on how to verify their authenticity. The paper accepts that these videos are abundant and necessary in an age driven by open access to information and commercial interests.

**Methods:**

The paper uses evidence from previous studies and observations of authors to propose the inclusion of YouTube in the health promotion toolkit of Ghanaian and African health systems.

**Results:**

The paper proposes the improvement of health literacy and patient-caregiver communication in preparation for an active role for YouTube as a health promotion tool.

**Conclusions:**

For patients, the paper recommends improved health literacy and communication with caregivers as an effective safety mechanism against misleading content. Caregivers are advised to accommodate patient views influenced by YouTube videos and be active participants in online spaces. Research on health literacy and effective patient-caregiver communication is recommended.

## 1 Introduction: YouTube and health promotion

Type the search term ‘when is blood pressure too high?’ on YouTube and you will have to choose from a long list of videos. There are video channels dedicated to health promotion and authors post everything from hypertension to diabetes, cancer, and nutrition (Clark et al., 2023, Gage-Bouchard et al., 2017). Health information on YouTube is enormous (Osman et al., 2022, Samuel et al., 2017). Some works like that of Drozd, Couvillon, and Suarez (2018) have recommended the use of YouTube as a health literacy tool and a part of public health promotion and information sources.

The recommendation is good news in Ghana, Africa, and the rest of the world for several reasons. Firstly, many adults spend significant portions of their day on social media and are likely to easily see such videos in their feeds. The US National Cancer Institute in the HINT 5 CYCLE 4 survey noted an increase in adults who consume health related content on YouTube from 39.7 % in 2020 to 58.9 % in 2022 (Mohamed & Shoufan, 2024). YouTube videos shared on TikTok, and other social media platforms may end up trending while sending important messages (Al-Maroof et al., 2021). Also, individuals who create this content do so without the rigorous and time-consuming processes that are sometimes involved with other publishing forms. The absence of such vetting allows the free posting of numerous health promotion videos about conditions which need urgent global attention such as diabetes, high blood pressure and obesity (Fernandez-Llatas et al., 2017). YouTube is advantageous because it allows for the demonstration of freedom of information and provides an open space to disseminate health promotion content. However, there are some risks including the possible commercial interests behind the videos and the possibility of harmful behavioral change decisions by patients (Osman et al., 2022, Guzman et al., 2020).

Through a review of recent peer-reviewed journals on health promotion and YouTube videos on Google Scholar, the authors discuss the growing consumption of YouTube in health care communication and its implications.

## 2 Credibility of YouTube health videos

YouTube videos can be created and posted by any user (Fernandez-Llatas., 2017). Trending videos don’t always come from popular media houses. It can come from a first-time user or a person with no following but when shared by others, it can gain traction and spread across the internet. This means ‘anyone’ can post content, including health related information. Unlike websites of prominent health centers like Mayo, Cleveland Clinic or local Ghanaian health professionals who audit all published information, YouTube information is not thoroughly audited before posting (Guzman et al., 2020). Viewers need to be aware of this and be guided when ‘consuming’ health related information. This is important because of the possibility of false information and hidden commercial interests behind videos (Osman et al., 2022).

While expertise must be a prerequisite for writing a book or publishing an article on a hospital website, on YouTube, that is not necessary. Viewers therefore need to ascertain whether the content creator is qualified to make those authoritative suggestions. Instructive videos which are designed to drive behavioral change and lifestyle adjustments need to be well scrutinized by viewers. Viewers do not always have the skills to verify whether the instructions are helpful or authentic (Guzman et al., 2020). As such effective patient-caregiver communication (Langford & Loeb, 2019) and health literacy (Feinberg et al., 2023) are recommended by this paper as solutions to this problem.

Caregivers should be ready to listen to patients when they discuss such content with them and can volunteer to watch videos online and recommend the best (Akakpo, 2022). Secondly, public health campaigns which drive health literacy will enable patients to verify information before they adhere to its instructions (Drozd et al., 2018). Whether the instructions in the video are good for the viewer can be subject to debate. However, effective patient-caregiver communication or health literacy can resolve these dilemmas (Langford and Loeb, 2019, Yom-Tov et al., 2016). In the next section the paper discusses the effectiveness of patient-caregiver communication and health literacy.

### 2.1 Patient-caregiver communication pattern

Caregivers are fully aware of the influence the internet has on patients (Langford & Loeb, 2019). Patients visit hospitals and reference videos or online content when discussing their health with caregivers. Many studies have confirmed this reality (Samuel et al., 2017) and advised caregivers to be accommodating (Drozd et al., 2018, Akakpo, 2022). This means caregivers should listen to the patients’ views and offer sound professional advice about the best place to seek health advice online (Langford and Loeb, 2019). A more credible form would be for caregivers to create content themselves and refer patients to their content or other credible online information (Gage-Bouchard et al., 2017). The planning of treatment and review of adherence regimes is best done between the patient and caregiver. However, tips about basic topics like diet, exercise or self-examination can be given with videos. Irrespective of caregivers’ opinions, patients are likely to use the internet to check their health, find other caregivers or advise other patients (Samuel et al., 2017). To reduce the risk of consuming misleading information, caregivers can recommend the best content to their patients.

### 2.2 Health literacy

With the abundance of information on YouTube, health literacy can be an effective ‘compass’ to guide patients when surfing for health information (Drozd et al., 2018). Health literacy has been identified as an effective tool for good health because it helps patients make timely decisions about their health (Feinberg et al., 2023). Health literacy further guides patients in their treatment adherence and receptiveness to prevention information (Yom-Tov et al., 2016). It is a skill which can help patients avoid misleading information on YouTube and avoid taking decisions without expert medical advice. Health literacy can guide patients to verify the authenticity of videos by evaluating the interests of the content creator and the urgency with which they must adhere to the content of these videos (Feinberg et al., 2023). A health literate person does not need constant coaching from caregivers and does not need to be retrained when a public health emergency arises (Jiang & Beaudoin, 2016). Rather, they possess the basic decision-making tools which allow them to vet health information and determine when they should visit a caregiver (Yom-Tov et al., 2016). As such, this skill should therefore be trained in all individuals because it can be a safety mechanism against misleading content.

## 3 Conclusion

As the volume of information continues to grow, channels multiply and access opens, risks will also expand (Guzman et al., 2020). To mitigate this while reaping much benefit, this paper is recommending effective patient-caregiver communication and patient health literacy. Global health issues like HIV/AIDS, Tuberculosis, and recent crises like Covid-19, have taught us the value of health system resilience (Feinberg et al., 2023). This resilience can be achieved when all stakeholders, including patients, are well informed and prepared. The internet is arguably the single biggest repository of world-wide information with significant daily growth in content (Fernandez-Llatas et al., 2017). This makes it an important tool for health promotion campaigns. Governments, health experts and patients can use this avenue to create and distribute health promotion information. Just as gathering people in a community hall or a school were effective, YouTube videos can also be effective tools for health behavior change (Drozd et al., 2018).

Conclusions from the paper have implications for researchers, caregivers, and patients. This is discussed in the next section.

## 4 Implications for Researchers, Caregivers, and patients

Well-researched and effective evaluation criteria which can be used by patients to quickly assess the credibility of health promotion videos on YouTube is needed. These criteria could be taught in schools, advertised in hospitals, or even promoted online. Research to guide caregivers on effective patient-caregiver communication especially regarding recommending the best avenues for health promotion information is also imperative.

Caregivers must accommodate information derived by patients from online sources. Health promotion can be achieved by recommending videos, providing tips to improve patient health literacy, and the creation of content by health providers themselves. The readiness of the patients to watch health promotion videos on YouTube can drive patient autonomy. Also, recommendations from caregivers will be more trustworthy for patients and more likely to fit their specific health needs. If patient-health literacy is trained, it will serve as an autonomous measure of safety for patients. Because of the adverse effects of unreliable health information on patient treatment, it is further incumbent on caregivers to promote patient health literacy.

To conclude, while it has been established that YouTube can be a safe source of quality health information, it is safest to discuss important behavioral change information with caregivers. In doing this, and to provide additional resources and information to guide patients in making informed health decisions, the health literacy of patient’s need to be improved. This will enable the right or authentic health videos to serve their purpose. Information is a key contributor to patient behavioral (lifestyle) change which drives the prevention and management of many health conditions. The internet is now an important source of this information but to use it effectively, patients need to communicate with their caregivers and improve their knowledge about important health topics.

## Funding

Funding was not secured for this project.

## CRediT authorship contribution statement

**Martin Gameli Akakpo:** Conceptualization, Investigation, Project administration, Supervision, Writing – original draft, Writing – review & editing. **Evelyn Owusu Roberts:** Conceptualization, Writing – original draft, Writing – review & editing.

## Declaration of competing interest

The authors declare that they have no known competing financial interests or personal relationships that could have appeared to influence the work reported in this paper.

## Data Availability

No data was used for the research described in the article.
